# Laminin-α4 Is Upregulated in Both Human and Murine Models of Obesity

**DOI:** 10.3389/fendo.2021.698621

**Published:** 2021-07-28

**Authors:** Anna Goddi, Alanis Carmona, Liesl Schroedl, Jeremy M. White, Matthew J. Piron, Avelino De Leon, Isabel Casimiro, Alexandria Hoffman, Maria A. Gonzalez Porras, Eric M. Brey, Matthew J. Brady, Ronald N. Cohen

**Affiliations:** ^1^Committee on Molecular Metabolism and Nutrition, The University of Chicago, Chicago, IL, United States; ^2^Section of Endocrinology, Diabetes, and Metabolism, The University of Chicago, Chicago, IL, United States; ^3^Pritzker School of Medicine, The University of Chicago, Chicago, IL, United States; ^4^Department of Biomedical Engineering and Chemical Engineering, The University of Texas at San Antonio, San Antonio, TX, United States

**Keywords:** laminins, extracellular matrix, basement membrane, adipose tissue, obesity, metabolic disease

## Abstract

Obesity affects nearly one billion globally and can lead to life-threatening sequelae. Consequently, there is an urgent need for novel therapeutics. We have previously shown that laminin, alpha 4 (*Lama4*) knockout in mice leads to resistance to adipose tissue accumulation; however, the relationship between *LAMA4* and obesity in humans has not been established. In this study we measured laminin-α chain and collagen mRNA expression in the subcutaneous white adipose tissue (sWAT) of mice placed on chow (RCD) or 45% high fat diet (HFD) for 8 weeks, and also in HFD mice then placed on a “weight loss” regimen (8 weeks HFD followed by 6 weeks RCD). To assess extracellular matrix (ECM) components in humans with obesity, laminin subunit alpha mRNA and protein expression was measured in sWAT biopsies of female control subjects (BMI<30) or subjects with obesity undergoing bariatric surgery at the University of Chicago Medical Center (BMI>35) both before and three months after surgery. Lama4 was significantly higher in sWAT of HFD compared to RCD mice at both the RNA and protein level (p<0.001, p<0.05 respectively). sWAT from human subjects with obesity also showed significantly higher *LAMA4* mRNA (p<0.01) and LAMA4 protein expression (p<0.05) than controls. Interestingly, even though LAMA4 expression was increased in both humans and murine models of obesity, no significant difference in *Lama4* or LAMA4 expression was detected following short-term weight loss in either mouse or human samples, respectively. From these results we propose a significant association between obesity and elevated LAMA4 expression in humans, as well as in mouse models of obesity. Further studies should clarify the mechanisms underlying this association to target *LAMA4* effectively as a potential therapy for obesity.

## Introduction

Obesity is an epidemic affecting 13% of people worldwide and contributing to more than $2 trillion in global economic impact (1, 2). Moreover, obesity can lead to the development of serious health conditions including cancer, hypertension, heart disease, and type 2 diabetes (3–5). A clear necessity arises for the development of adipose targeted therapies to ameliorate metabolic dysfunction and reduce overall adiposity. In recent years, much has been learned about adipocyte biology, in particular the intracellular signaling pathways and transcription factors that mediate its function. However, less attention has been paid to the adipose microenvironment, with the exception of significant focus on macrophage infiltration that occurs in the setting of obesity. Recent work suggests that the extracellular matrix (ECM) itself considerably affects adipocyte biology.

The ECM is a network of macromolecules that contributes to cell support, migration, and signaling (6). Three major classes of ECM biomolecules have been identified including structural proteins (collagen, elastin), specialized proteins (laminin, fibronectin), and proteoglycans (7). Of these proteins, most emphasis in the field of adipocyte biology and obesity has been placed on general fibrosis and collagen which accounts for the largest proportion of the stromal ECM. Type 4 and type 6 collagen are essential components of the adipocyte microenvironment but have also been implicated in obesity and related inflammatory phenotypes. In humans, collagen type IV alpha 1 (COL4A1) expression is significantly elevated in adipocytes from obese sWAT and furthermore, this expression diminishes 6 months after bariatric surgery (8). Collagen type VI alpha 3 (COL6A3) is enriched around adipocytes in mice, however there is conflicting evidence as to its association with obesity in humans. Khan et al., 2009 report that COL6A3 expression is elevated during states of metabolic stress and adipose dysregulation in mice and humans (9). Conversely, McCulloch et al., 2015 observed reduced COL6A3 expression in adipose of humans with obesity and suggest that COL6A3 is not the predominant collagen in human adipose tissue, indicating that such ECM proteins may serve different roles in murine and human adipose (10). It is also evident that the composition of the ECM can be detrimental to adipocyte function, yet very few studies have looked in depth at the relationship between non-collagen basement membrane proteins and obesity, especially in human adipose tissue.

In particular, laminins are heterotrimeric basement membrane proteins each composed of an α, β, and γ chain. There are currently sixteen isoforms identified and which are distributed in a tissue-specific manner (11). The laminin-α chains, which determine tissue specific expression patterns, contain differing numbers of laminin G-like (LG) domains in the C-terminus allowing for interaction with different types of receptors, such as integrins, syndecans, and dystroglycans (12). The LAMA4 chain differs from other laminin-α chains in that it contains no laminin N-terminal (LN) globular domain or laminin IV (LF) domain, and merely four laminin-type epidermal growth (EGF) factor-like (LE) domains (11). Laminin-α3A, a splice variant of laminin-α3, is the only other laminin-α chain with a similarly truncated N-terminus. This N-terminus short arm of laminin-α chains has been previously shown to play a role in basement membrane assembly and organization through interactions with other ECM components (11).

The LAMA4 chain is known to be highly upregulated during adipogenesis and has recently been suggested to engage integrin α7 on the adipocyte surface (13–15). As with other laminin chains, LAMA4 is implicated in ECM remodeling in several tissue types including muscle and various tumor types, however it is only just beginning to be associated with adipose tissue remodeling and dysfunction in obesity (16, 17). Moest et al., 2013 show LAMA4 deposition at the adipocyte surface is increased in both diet-induced-obesity (DIO) and genetic ob/ob mouse models compared to controls (18). Our group has demonstrated that mice deficient in *Lama4* are protected from DIO and exhibit improved insulin sensitivity (19). In non-diabetic humans with obesity, LAMA4 levels were found to be increased in the secretome of visceral WAT (vWAT) adipocytes compared to sWAT, however the opposite trend has been reported in mice (18, 20).

While these results suggest that LAMA4 may be an important regulator of adipocyte function, its clinical relevance to various models of obesity remains poorly characterized. In this study we aim to describe the relationship between LAMA4 and obesity in both mouse and human models and determine the effect of weight loss on LAMA4 expression. We analyzed laminin and collagen expression in the sWAT of mice following high fat diet (HFD) feeding and found that the expression of *Lama2*, *Lama4*, and several collagen subunits were significantly upregulated in the HFD fed mice. Of the laminin-α chains, only *Lama4* expression strongly correlated with weight. We then investigated laminin expression in humans using sWAT samples from female control subjects (BMI<30) and female subjects with obesity (BMI>35) undergoing bariatric surgery (baseline vs. 3 months post-surgery) and discovered that the expression of LAMA4 was significantly upregulated in the obese sWAT at both the mRNA and protein level. Interestingly, short-term weight-loss in both humans and mice did not result in a significant change in LAMA4 expression. We highlight an important relationship between LAMA4 and obesity in both mice and humans, suggesting that laminins play a critical role in obesity development in human subjects.

## Materials and Methods

### Animal Care and Diet Studies

Animal procedures and numbers were approved by the University of Chicago Institutional Animal Care and Use Committee. WT male and female C57BL/6J mice were fed either regular chow diet (RCD, Teklad 2918; Harlan Laboratories) or 45% high fat diet (HFD, Teklad custom diet TD.06415; Harlan Laboratories) ad libitum and housed at room temperature. Beginning at 7 weeks of age, mice were weighed weekly until completion of the study. Two separate dietary studies were performed: RCD vs HFD for 8 weeks and HFD vs “weight loss” group where mice were fed HFD for 8 weeks and then switched to RCD for another 6 weeks (14 weeks total of dietary study). Mice were humanely killed at 16 or 22 weeks of age, respectively. Tissues were collected and snap frozen in liquid nitrogen and stored at -80°C.

### Human Samples and Clinical Parameters

10 non-diabetic female subjects with obesity undergoing laparoscopic sleeve gastrectomy (BMI>35) and 3 female control subjects (BMI<30) between the ages of 20 and 55 were voluntarily enrolled by the Center for the Surgical Treatment of Obesity at the University of Chicago. Eligibility requirements restricted subjects with diabetes or on medications for diabetes or that would alter glucose metabolism from participating in the study. Subcutaneous fat samples were collected *via* a needle biopsy 2 weeks prior to and 12-13 weeks post bariatric surgery according to a protocol modified from that of Carswell et al. (21, 22).

### RNA Extraction

Tissue was homogenized using a Bullet Blender^®^ at 4°F (Next Advance PINKE1, BBX24B) or a dispersion-based homogenizer (VWR VDI 12) for human and mouse samples, respectively. RNA was isolated using the E.Z.N.A Total RNA Kit II (Omega Biotek; R6934) following the manufacturer’s instructions.

### Quantitative Real-Time PCR

The RNA samples were reverse transcribed using Quanta QScript Master Mix (VWR; 95048) and 500 ng RNA per 20μL sample reaction volume. Quantitative Real-Time PCR was performed with SYBR green using a Bio-Rad CFX Connect Real-Time PCR Detection System. Primers were purchased from IDT or Qiagen and can be found in **Supplemental Table 1**. For murine samples, *Gapdh* was used as a reference gene. For human samples the composite of *GAPDH*, *YWHAZ*, and *RPL13A* was used to control for total mRNA as previously described (22). Gene expression was evaluated by ddCT methods.

### Immunoblotting

Samples were lysed using cold 1X RIPA buffer (EMD Millipore; 20-188) containing 1X phosphatase and protease inhibitor cocktail tablets (Roche) and a dispersion-based homogenizer (VWR VDI 12). After incubating on ice for 30 minutes, samples were briefly sonicated (Sonics Vibra-cell) and spun at 1,000xg for 10 minutes at 4°C to separate the lipid layer. The supernatant was collected and spun again at 12,000xg for 10 minutes at 4°C and supernatant from this spin was collected and stored at -80°C. Protein concentration was determined using the Pierce BCA Protein Assay Kit (Thermo Fisher Scientific; 23227). Samples were diluted in water and 4X Laemmli Sample Buffer (Bio-Rad; 1610747) and run on 4-15% SDS-PAGE Mini-PROTEAN TGX gels (Bio-Rad) then transferred to Immobilon-P PVDF membranes (EMD Millipore). Membranes were blocked with 5% PhosphoBLOCKER™ Blocking Reagent (Cell Biolabs, Inc; AKR-103) in TBST for LAMA4 probing, or 5% Non-fat dry milk (LabScientific; M0841) in TBST for other antibodies for 1 hr. Blots were incubated overnight at 4°C in 1% blocking solution with LAMA4 antibody (Invitrogen; mouse mAb, MA5-24650) or β-Actin (CST; Rabbit mAb, 4970). Membranes were then incubated with IRDye secondary antibodies (LI-COR) for 1 hr. Immunodetection was performed using near-infrared Odyssey CLx System (LI-COR). Analysis was performed issuing the Image Studio Software (LI-COR).

### Immunofluorescence

Tissue was fixed in 10% formalin, paraffin embedded, and sectioned at 5μm thickness by a microtome at the University of Chicago Human Tissue Resources Center. Sections were baked for 60 minutes at 60°C, deparaffinized, and rehydrated in xylenes and alcohol. Heat induced epitope retrieval was performed with citric acid-EDTA buffer pH 6.2 as recommended by the manufacturer of the LAMA4 antibody. Sections were blocked in 10% donkey serum (Abcam ab7475) and incubated with primary antibody (anti-laminin alpha 4, Novus NBP2-42393, 1:300 dilution) overnight at 4°C. An Alexa Fluor-488 conjugated secondary antibody was added for 1 hour at room temperature (Abcam ab150113). Following incubation, sections were washed, stained with DAPI (Invitrogen S36939), and sealed. Images were taken using Fixed-DSU Confocal at the University of Chicago Integrated Light Microscopy Core and quantification of fluorescent signal was determined using ImageJ. All images were processed equally and in an unbiased manner to remove intracellular LAMA4 signal and background noise before signal quantification and analysis. Macro code for ImageJ analysis can be found in the Supplementary Materials file.

### Adipose Derived Stem Cell Isolation and Differentiation

Primary adipose derived stem cells were isolated from 14-17 week old WT male mice as previously described (23). Cells were expanded and plated into 6-well plates for experiments, and subsequently differentiated to white adipocytes following a previously published protocol (24). Induction medium containing complete medium supplemented with 17nM insulin, 60uM indomethacin, 0.1uM dexamethasone, and 250uM isobutylmethylxanthine was added to cells to initiate differentiation. On day 2 cells were given maintenance medium containing complete medium with 17nM insulin. Following this refeeding occurred every other day with complete medium (10% fetal bovine serum, 1% pen/strep, DMEM/F-12).

### LN411 Coating

6-well plates were coated with 0ug or 10ug of recombinant laminin-411 protein (BioLamina LN411) in DPBS per well according to manufacturer instructions. Plates were incubated at 37°C for 2 hours and the solution was aspirated before cell seeding. For all experiments involving LN411 coating, primary adipose derived stem cells were differentiated to white adipocytes as described. Adipocytes reached maturity by day 8 and were collected for RNA.

### Fatty Acid Treatment

On day 6 and 8 of differentiation, adipocytes were treated with 1.5mM BSA (Sigma A8806) in DPBS as the vehicle, 0.25mM Oleic Acid (Sigma O3008), or 0.25mM Palmitic Acid (Caymen Chemical; 29558). Cells were collected on day 10 of differentiation. The majority of studies utilized oleic acid to stimulate lipid loading as palmitic acid did not lead to enhanced lipid loading or increases of adipogenic gene expression.

### Lipolysis

Basal lipolysis of cells cultured on LN411 was quantified using the Abcam lipolysis assay kit (ab185433) with volumes adjusted to fit a 24-well plate format. Cells were washed in lipolysis wash buffer and then incubated with lipolysis assay buffer for 2.5 hours. Glycerol concentration in the assay buffer was determined by absorbance readings at 570 nm after incubation with the kit Reaction Mix for 30 minutes. Glycerol was then normalized to cellular protein content using the Pierce BCA Protein Assay Kit (Thermo Fisher Scientific; 23227).

### Statistics

Statistical tests for the different studies were computed as follows: for mouse studies where number of mice was essentially equal between groups and variance was expected to be similar, a student’s t-test was performed for all data. In all human sample RNA analyses, statistical comparisons between control (n=3) and obese pre-surgery (n=10) were assessed with a Welch’s unequal variances t-test, while comparisons between obese pre-surgery (n=9) and post-surgery (n=9) were assessed with a paired sample t-test, as RNA was obtained for both groups from the same set of subjects. In experiments involving LAMA4 staining of fixed human sWAT tissues where both pre (n=9) and post-surgery (n=6) samples could not be obtained from all subjects, a student’s t-test was performed. In all cases, p<0.05 was considered significant.

## Results

### Laminin and Collagen Expression in a Mouse Model of Obesity

**Figure 1:**
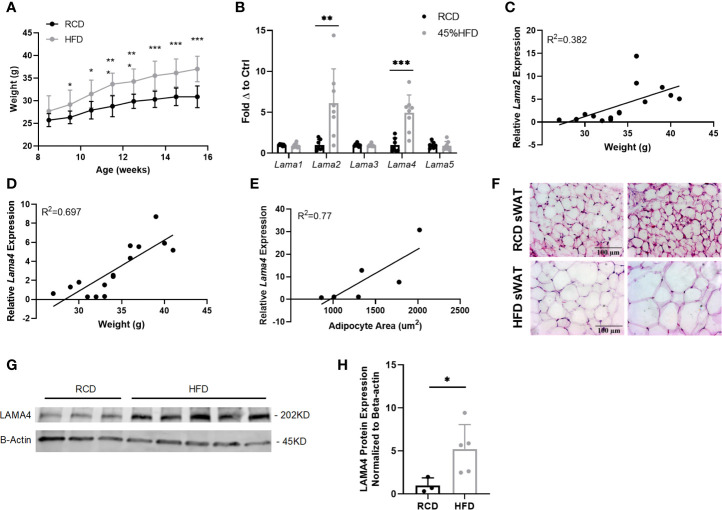
Gene expression of laminin-α chains in sWAT of HFD fed mice. **(A)** Average weekly weights of mice on regular chow diet (RCD) and 45% High Fat Diet (HFD) for 8 weeks. RCD (n=7), HFD (n=8). (*,**,***) indicates p < 0.05, 0.01, 0.001, respectively. Data are Means ± SD. **(B)** Laminin-α chain mRNA expression in sWAT of mice after 8 weeks of dietary study normalized to the average of RCD control group. RCD (n=7), HFD (n=8). **(C)** Relative *Lama2* mRNA expression normalized to RCD group graphed against weight (grams) for each mouse in 8-week dietary study (n=15). Simple linear regression analysis. **(D)** Relative *Lama4* mRNA expression graphed against weight (grams) for each mouse in 8-week dietary study (n=15). Simple linear regression analysis. **(E)** Relative *Lama4* mRNA expression graphed against adipocyte area (um^2^) for one cohort of HFD and RCD mice (n=6). Simple linear regression analysis. **(F)** Representative images of H&E stained sWAT tissue sections of HFD and RCD mice after 8 weeks of dietary study. All images are 40X magnification. **(G)** Protein expression of LAMA4 and loading control B-actin in sWAT of mice placed on RCD or 45% HFD for 8 weeks was assessed by western blot. Original blot images can be found in the supplementary file. **(H)** LAMA4 signal from the western blot was quantified and normalized to the loading control signal. Values are shown as fold changes in comparison to the average of the RCD group. RCD (n=3), HFD (n=5).

To study the relationship between *Lama4* and obesity, we placed 8-week-old WT male mice on regular chow diet (RCD) or 45% high fat diet (HFD) for 8 weeks. Preliminary dietary studies showed that female mice placed on HFD did not gain significant weight during the dietary study timeline and therefore were not a good model to study the association of *Lama4* and obesity (**Supplemental Figure 1**). The male mice on HFD gained considerably more weight than the RCD group (**Figure 1A**). At 16 weeks of age we assessed the mRNA expression of laminin-α chains in adipose depots by quantitative real-time PCR (qrtPCR). Only subcutaneous WAT showed significant differences in laminin-α chain expression, while epididymal WAT and brown adipose tissue showed no significant differences, which informed our decision to focus on sWAT for the duration of the murine study (**Supplemental Figure 2**).

The expression of both *Lama2* and *Lama4* was significantly higher in sWAT of the HFD group compared to the RCD group, by 5.9 and 4.0-fold respectively (**Figure 1B**). *Lama2*, while predominantly expressed in muscle tissue and implicated in diseases of muscular dystrophy has also recently been shown to inhibit osteogenesis and promote adipogenesis of mesenchymal stem cells (MSCs) *via* the hedgehog signaling pathway (25). When relative *Lama2* expression was compared to individual mouse weights at 16 weeks we found only a weak correlation (R^2^ = 0.38) suggesting that *Lama2* expression may not directly tie to adipose tissue mass in mice (**Figure 1C**). We did however identify a strong positive correlation between relative *Lama4* expression and weight (R^2^ = 0.70) and between relative *Lama4* expression and adipocyte area (R^2^ = 0.77) (**Figures 1D–F**).

The observed upregulation in *Lama4* was confirmed at the protein level by western blotting. Protein lysates from sWAT of HFD mice showed a 5-fold significant elevation of LAMA4 protein in comparison to RCD samples (**Figures 1G, H**). These results indicate that, of all laminin-α chains, LAMA4 appears to be strongly related to subcutaneous white adipose accumulation in male mice.

**Figure 2:**
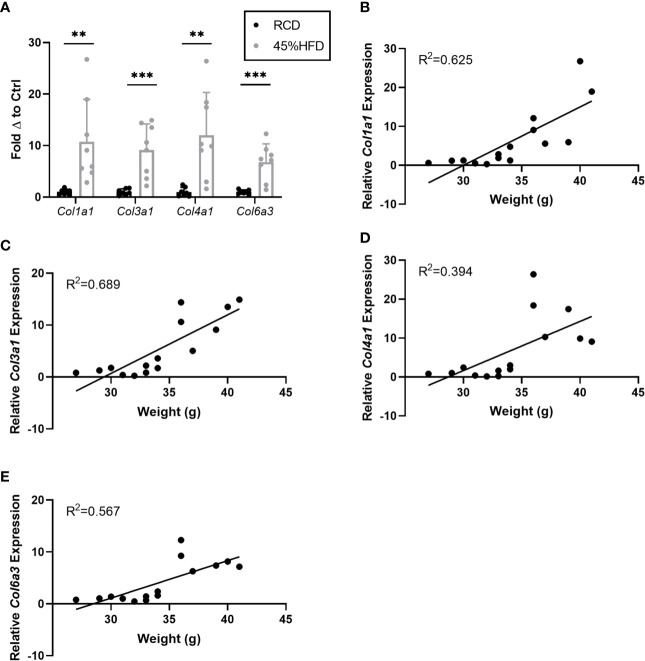
Gene expression of collagen species in sWAT of HFD fed mice. **(A)** Collagen species mRNA expression in sWAT of mice after 8 weeks of dietary study normalized to the average of RCD control group. RCD (n=7), HFD (n=8). (**,***) indicates p < 0.01, 0.001), respectively. Data are Means ± SD. **(B–E)** Relative *Col1a1* mRNA expression normalized to RCD group graphed against weight (grams) for each mouse in 8-week dietary study (n=15). Simple linear regression analysis.

In addition to laminin chains we also assessed the expression of several collagen subunits known to be associated with obesity in mice. We found that all collagen subunits tested (*Col1a1*, *Col3a1*, *Col4a1*, and *Col6a3*) were significantly upregulated in the sWAT of HFD mice compared to controls (**Figure 2A**). *Col1a1* and *Col3a1* showed the greatest positive correlation with weight (R^2^ = 0.625 and 0.689, respectively) (**Figures 2B–E**).

### *In Vitro* Relationship of Lama4 and Adipocyte Function

**Figure 3:**
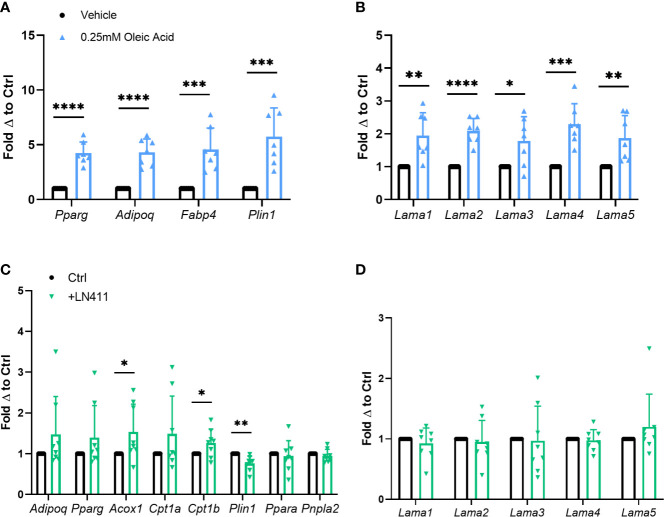
Relationship of *Lama4* to lipid storage and metabolism in *vitro*. **(A)** Gene expression of adipogenic markers in primary murine pre-adipocytes treated with vehicle or 0.25mM Oleic Acid during differentiation. Vehicle (n=7), Oleic Acid (n=7). (*,**,***,****) indicates p < 0.05, 0.01, 0.001, 0.0001 respectively. Data are Means ± SD. **(B)** Laminin-α chain gene expression in primary differentiated murine adipocytes treated with vehicle or 0.25mM Oleic Acid during differentiation. Vehicle (n=7), Oleic Acid (n=7). **(C)** Expression of genes involved in lipid metabolism, oxidation, and adipogenesis in primary murine differentiated adipocytes grown on culture plates coated with 0ug (n=8) or 10ug of LN411 (n=8). **(D)** Laminin-α chain gene expression in primary murine differentiated adipocytes grown on culture plates coated with 0ug (n=8) or 10ug of LN411 (n=8).

*In vitro* adipocyte studies were performed assessing the effect of heightened lipid loading *via* oleic acid treatment on *Lama4* expression levels. Previous studies show that treatment of 3T3-L1 adipocytes with oleic but not palmitic or stearic acid induces significant lipid loading and expression of adipogenic genes (26). Primary murine adipose derived stem cells (ADSCs) isolated from WT male mice were differentiated to mature white adipocytes and treated with 0.25mM fatty acid or vehicle for the last 4 days of the 10-day differentiation timeline. Expression of adipogenic markers such as peroxisome proliferator activated receptor gamma (*Pparg*), adiponectin (*Adipoq*), fatty acid binding protein 4 (*Fabp4*), and perilipin 1 (*Plin1*) were significantly upregulated in the oleic acid treated group (**Figure 3A**). Heightened lipid loading, as measured by semi-quantitative Oil Red O staining, was observed in the oleic acid treated group as well, but not in the palmitic acid treated group (**Supplemental Figures 3B, C**). There was around a 2-fold increase in expression of laminin-α chains in the cells treated with the oleic acid, with *Lama2* and *Lama4* being the most statistically significant (**Figure 3B**). We observed no elevation in laminin-α chain mRNA expression in the cells treated with palmitic acid (**Supplemental Figure 3A**).

We next studied changes in lipid metabolism and fatty acid β-oxidation in response to growth on recombinant laminin-411 (LN411) coated wells. The LN411 trimer contains the LAMA4, laminin beta 1 (LAMB1), and laminin gamma 1 (LAMC1) chains. Primary murine ADSCs differentiated to white adipocytes on plates coated with 10ug of LN411 displayed significantly decreased gene expression of *Plin1* and increased expression of acyl-CoA oxidase 1, palmitoyl (*Acox1*) and carnitine palmitoyltransferase 1b (*Cpt1b*) compared to those grown on uncoated wells (**Figure 3C**). Expression of related genes such as patatin-like phospholipase domain containing 2 (*Pnpla2*, previously known as adipose triglyceride lipase, *Atgl*) and peroxisome proliferator activated receptor alpha (*Ppara*) showed no significant changes while *Adipoq* and *Pparg* expression trended upwards in the LN411 treated group. No changes in laminin-α chain expression was observed, suggesting that the results are most likely due to LN411 mediated changes and not resultant from endogenous ECM compositional alterations (**Figure 3D**).

To further investigate changes in lipid metabolism based on the previous results we assessed lipolysis rate in cells grown on 10ug of LN411. The LN411 group displayed slightly elevated basal lipolysis rate compared to control cells (p=0.05) (**Supplemental Figure 4A**). Interestingly, lipid loading as measured by semi-quantitative oil red o staining revealed a small but significant elevation in the LN411 group as well (**Supplemental Figures 4B, C**).

### Laminin and Collagen Expression in Human Obesity

**Figure 4:**
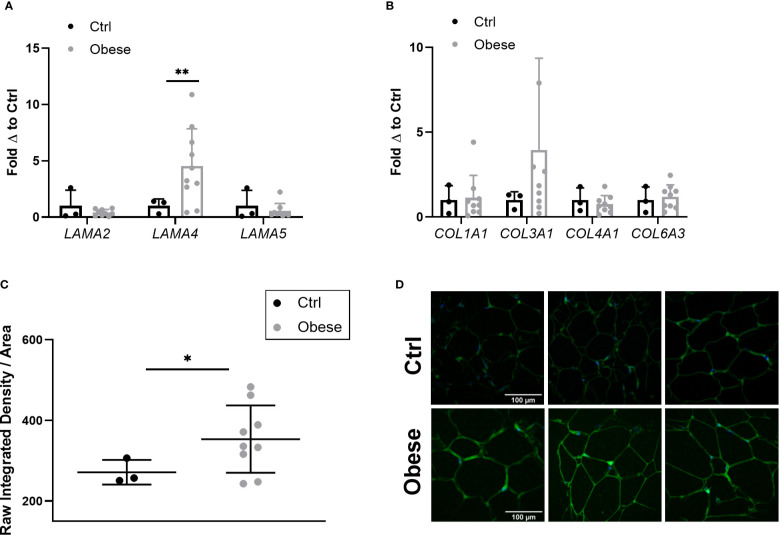
Expression of laminin-α chains and collagen species in sWAT of human subjects. **(A)** Laminin-α chain mRNA expression in sWAT biopsies of female control (BMI<30) and obese (BMI>35) subjects normalized to average of control subjects. Control (n=3), Obese (*LAMA2*, *LAMA5* n=9, *LAMA4* n=10). (*,**) indicates p < 0.05, 0.01, respectively. Data are Means ± SD. **(B)** Collagen species mRNA expression in sWAT biopsies of human subjects normalized to average of control subjects. Control (n=3), Obese (n=9). **(C)** Density of extracellular LAMA4 as determined by raw integrated density signal over total area of signal in fixed sections of sWAT from control and obese subjects. Control (n=3), Obese (n=9). **(D)** Representative images of extracellular LAMA4 immunofluorescence staining from control and obese sWAT. LAMA4 (in green) and DAPI (in blue). All images are 40X magnification.

As *Lama4* expression was significantly augmented in HFD fed mice, we wanted to understand if this trend applied to human obesity. We were able to obtain a set of sWAT RNA samples from female control subjects (BMI<30) and non-diabetic subjects with obesity (BMI>35) undergoing bariatric surgery (subject characteristics listed in **Table 1**) from an ongoing study at the University of Chicago investigating bariatric surgery and circadian rhythms in females. We measured the gene expression of several laminin-α chains known to be expressed in adult adipose tissue. The sWAT samples from subjects with obesity pre-surgery displayed 4-fold greater *LAMA4* expression than the control subjects (p<0.01) (**Figure 4A**). We found no change in the mRNA expression levels of *LAMA2* or *LAMA5* (**Figure 4A**). In contrast to our results of the mouse studies, we were unable to detect a significant difference in the expression of *COL1A1*, *COL3A1*, *COL4A1*, and *COL6A3* between the two groups (**Figure 4B**). This may arise from the control group containing some overweight but not obese subjects (25<BMI<30) where collagen species may already be highly expressed.

**Table 1:** Human Study Subject Characteristics (Gene Expression Experiments).

Subjects	Control	Obese pre-surgery	Obese post-surgery
	N = 3	N = 10	N = 10
**Weight (kg)**	64.13 (± 8.12)	124.95 (± 13.33)	103.48 (+ 12.19)
**Height (cm)**	161.76 (± 7.35)	166.85 (+ 6.34)	166.53 (+ 6.50)
**BMI (kg/m^2)**	24.53 (± 2.59)	44.95 (+ 5.12)	37.41 (+ 4.86)
**Age**	39 (± 16.52)	36 (+ 8.96)	36.2 (+ 8.66)
**HbA1c (%) at Screening**	5.43 (± 0.30)	5.54 (± 0.36)	5.54 (± 0.36)
**Fasting Glucose (mg/dL)**	87.55 (± 21.61)	87.38 (± 5.11)	75.55 (± 6.23)
**Fasting Insulin (pmol/L)**	56.52 (± 9.57)	121.57 (± 78.65)	29.37 (± 16.12)

IVGTTs were not performed or could not be safely completed for some subjects in the study. For obese pre-surgery, fasting glucose and insulin is shown for 7/10 subjects, and for obese post-surgery it is shown to 6/10 subjects.

Additionally, we obtained fixed adipose tissue sections from a portion of the control subjects and subjects with obesity (study subjects characteristics for immunofluorescence experiments can be found in **Supplementary Table 2**). We verified by immunofluorescence staining that extracellular LAMA4 protein was also significantly increased in the subjects with obesity pre-surgery compared to the controls (p<0.05) (**Figures 4C, D**). The density of LAMA4 in the ECM of obese adipose was about 30% greater than in control adipose, suggesting that the adipocytes in sWAT of subjects with obesity do in fact produce and deposit more LAMA4 than adipocytes from subjects without obesity.

### LAMA4 Expression Does Not Change Following Weight Loss

**Figure 5:**
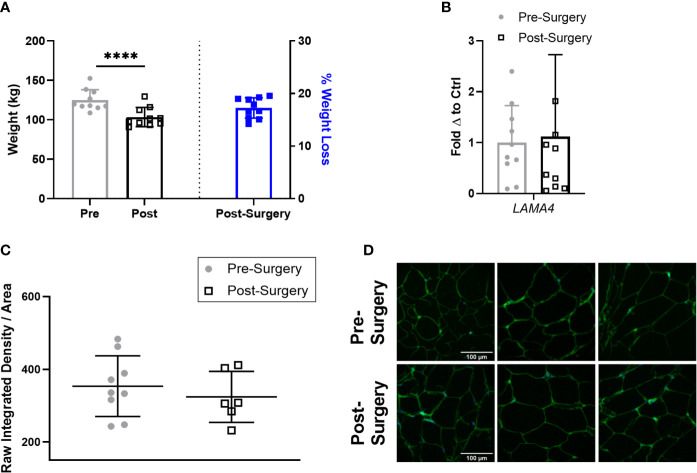
LAMA4 expression does not change following short term weight loss in humans with obesity. **(A)** Weight (kg) of human subjects before and 3 months post-surgery shown on left y-axis [(****) indicates p < 0.0001] and % of weight loss 3 months post-surgery is shown on right y-axis. Data are Means ± SD. **(B)** *LAMA4* mRNA expression in sWAT biopsies of obese subjects pre-surgery and 3 months post-surgery, normalized to average of pre-surgery subjects. Pre-surgery (n=9), Post-surgery (n=9). **(C)** Density of extracellular LAMA4 as determined by raw integrated density signal over total area of signal in fixed sections of sWAT from obese subjects pre-surgery and 3 months post-surgery. Pre-surgery (n=9), Post-surgery (n=6). **(D)** Representative images of extracellular LAMA4 immunofluorescence staining from obese pre-surgery and post-surgery subjects. LAMA4 (in green) and DAPI (in blue). All images are 40X magnification.

Next, we compared the expression of LAMA4 in the subjects with obesity 3 months after bariatric surgery to determine if short term weight loss could reverse the observed upregulation of LAMA4. At this stage following bariatric surgery the subjects with obesity had lost a small but statistically significant percentage of weight compared to pre-surgery, averaging around 17% weight loss (**Figure 5A**). However, we found that sWAT post-surgery samples had similar levels of *LAMA4* gene expression on average to the samples taken pre-surgery (**Figure 5B**). We observed no difference in density of extracellular LAMA4 between pre-surgery and post-surgery fixed adipose sections as determined by immunofluorescence staining (**Figures 5C, D**). Our findings indicate that short term weight loss in humans does not lead to consistent decreases in levels of LAMA4 expression.

**Figure 6:**
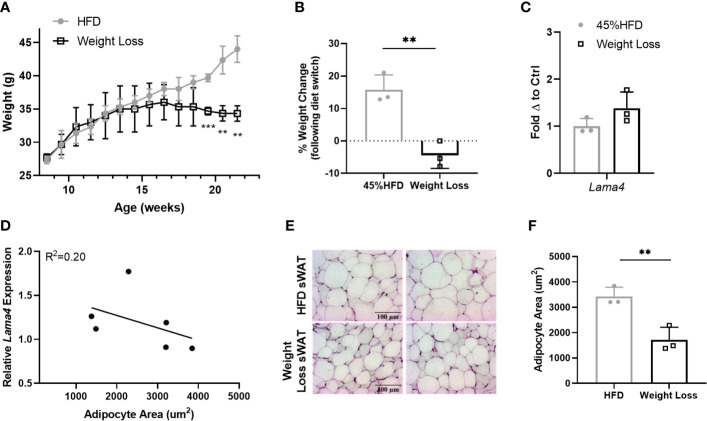
LAMA4 expression does not change following HFD feeding reversal in mice. **(A)** Average weekly weights of mice on 45% HFD for 14 weeks (HFD) or 45% HFD for 8 weeks and then RCD for 6 weeks. HFD (n=3), Weight Loss (n=3), (**,***) indicates p < 0.01, 0.001, respectively. **(B)** Weight change shown as percentage from timepoint of diet switch (16 weeks of age) until end of dietary study (22 weeks of age). Data are Means ± SD. **(C)** *Lama4* mRNA expression in sWAT of mice in HFD (n=3) or Weight Loss (n=3) group. **(D)** Relative *Lama4* mRNA expression graphed against adipocyte area (um^2^) (n=6). Simple linear regression analysis. **(E)** Representative images of H&E stained sWAT tissue sections of HFD and Weight Loss group mice after 14 weeks of dietary study. All images are 40X magnification. **(F)** Adipocyte area (um^2^) in sWAT of mice in HFD (n=3) and Weight Loss (n=3) group.

Following these results, we were interested to investigate *Lama4* expression following weight loss in a more controlled manner in mice. We placed two groups of 8-week -old WT mice on 45% HFD for 8 weeks and then switched one group to RCD for another 6 weeks while the other continued to receive HFD. The difference between the Weight Loss group (switched back to RCD) and HFD group became larger over the course of the 6-week diet period and by 22 weeks the Weight Loss group was significantly lower in weight than the group that remained on HFD (**Figure 6A**). From the time at which the diet was switched until the end of the study, the Weight Loss group lost on average around 5% of their body weight while the HFD group gained roughly 15% more weight (**Figure 6B**). We observed no difference in sWAT *Lama4* expression between the groups, indicating that reversal of HFD feeding and a small amount of weight loss, at least in a short timeframe, does not result in reduced *Lama4* expression levels (**Figure 6C**). In comparing the HFD and Weight Loss group, *Lama4* expression levels did not correlate strongly to adipocyte area, although the Weight Loss group did display significantly smaller adipocyte area in sWAT than the HFD group (**Figures 6D–F**).

## Discussion

The adipocyte microenvironment is an integral part of adipocyte functioning and in recent years has been further implicated in adipose dysfunction occurring in the obese state. However, how non-collagen ECM components such as laminins contribute to adipocyte signaling and response in this context remains poorly studied. In this investigation we characterize the relationship between obesity and LAMA4 in both mice and humans and illustrate the important role of this ECM protein in adipose tissue across models. We demonstrate a parallel upregulation of *Lama4* in male mice fed HFD and *LAMA4* in female human subjects with obesity compared to lean controls and verify that this upregulation is reflected at both the mRNA and protein level. We also conclude that short-term weight loss in DIO mice and human subjects with obesity does not downregulate LAMA4 expression. Overall, these findings describe a significant association between LAMA4 and obesity in humans.

LAMA4 expression is significantly upregulated during adipogenesis and has been linked to adiposity in previous studies. Moest et al., 2013 have established that LAMA4 is elevated in the sWAT and vWAT of DIO and ob/ob mouse models (18). Furthermore, our group has shown that the silencing of *Lama4* in mice leads to the reduced adipose mass accumulation and protection from obesity on a HFD (19). However, the applicability of this trend to humans was undetermined. In human obesity, LAMA4 has only been investigated to the extent of depot specific comparisons. Roca-Rivada et al., 2015 report heightened LAMA4 in secretomes of adipocytes from vWAT compared to sWAT in non-diabetic human subjects with obesity, however no control subjects were included for comparison (20). The results from our study demonstrate that sWAT *Lama4* expression is elevated by 4-fold in a DIO mouse model and positively correlates with weight and adipocyte area. Similarly, in humans, *LAMA4* expression increased by 4-fold in obese sWAT samples compared to controls. While *Lama2* was also upregulated in HFD fed mice, the expression was not found to correlate strongly with weight and was not upregulated in the sWAT of human subjects with obesity. While interesting and deserving of further investigation in mice, there appears to be no obvious association at present between *LAMA2* and obesity in humans. The unique elevation of *LAMA4* in obese sWAT suggests that this chain assumes a specialized role in the adipocyte microenvironment during obesity.

It should be noted that direct sex comparisons were not made in the course of this study. In line with previous studies showing that sexual dimorphisms exist with regards to DIO in mice, we found that female mice placed on HFD did not gain significant weight or become obese during the 8 week study period (27). Therefore, LAMA4 could not be studied in the context of DIO. Additionally, we obtained our human adipose samples from an ongoing bariatric surgery study examining outcomes in female subjects, and so we were not able to investigate this association in male subjects. However, it is noteworthy that we observe parallel findings in both species, although different sexes. Future investigations should compare LAMA4 expression in male and female subjects with obesity to determine if there are any differences based on sex.

Additionally, while we focused our investigation on subcutaneous adipose tissue in this study, it is not yet clear how transferrable insights from murine sWAT are to human abdominal sWAT function (28). Some studies suggest that gene expression for certain adipose functions in murine sWAT is more similar to human vWAT (29). Our results indicate promise that LAMA4 may serve similar roles in murine sWAT and human sWAT, however further verification may be needed to assess therapeutic potential of regulating laminins in human adipose depots. Other limitations of this study included the housing of mice at room temperature conditions rather than thermoneutrality. While this could have contributed to heightened energy metabolism compared to conditions at thermoneutrality, both dietary groups were housed at this same condition and thus could be considered comparable.

While it is not fully understood what role LAMA4 may play in aggravating obesity in humans, it is likely that LAMA4 is both deposited in elevated levels in response to obesity and can influence adipocyte function and pathways relating to lipid metabolism. Our results indicate that in a simulation of HFD feeding *in vitro*, laminin-α chains are significantly upregulated, suggesting that a heightened lipid burden stimulates laminin expression in adipocytes. Furthermore, cells differentiated to white adipocytes in the presence of LN411 showed diminished *Plin1* expression. PLIN1, located on lipid droplets, inhibits lipolysis and is downregulated in adipose of subjects with obesity (30). Our results suggest some involvement of LAMA4 in regulating PLIN1 levels, and thus excessive LAMA4 may contribute to elevated lipolysis and circulating non-esterified fatty acids which is detrimental to systemic insulin sensitivity. The results of the lipolysis assay showing enhanced lipolysis rates in LN411 cells support these findings.

Furthermore, genes related to β-oxidation were altered in response to LN411 treatment. *Cpt1b* is involved in mitochondrial β-oxidation and was significantly upregulated in cells differentiated on LN411. sWAT *CPT1A* expression has previously been positively correlated with BMI in humans, however neither isoform is very abundant in adipose tissue so it would be interesting to understand what role LAMA4 might play in CPT1 regulation in other tissues such as muscle (31). *Acox1*, which codes for the first enzyme in the peroxisomal β-oxidation pathway, was also found to be significantly upregulated in the LN411 samples. *Acox1* has been previously studied in the context of obesity as mice deficient in *Acox1* exhibit resistance to DIO through sustained *Ppara* activation (32). It is possible that LAMA4 may influence energy balance through a mix of genes involved in metabolism. While outside of the scope of this study, future investigations should focus on identifying specific relationships between LAMA4 and these metabolic pathways in adipocytes.

Lastly, in addition to investigating LAMA4 in obesity, we studied the effect of weight loss on expression of LAMA4 in sWAT. We found that short-term weight loss was not sufficient to reduce LAMA4 expression from the high levels seen in HFD fed mice or human subjects with obesity. This could be due in part to the duration of weight loss investigated in this study, and it is possible that longer periods of weight loss could eventually lead to a downregulation of LAMA4. As LAMA4 is implicated in worsening metabolic behavior, it will be important for future studies to assess if LAMA4 remains unchanged following other types and durations of weight loss.

## Conclusion

Although preliminary murine studies have suggested that obesity correlates with increased LAMA4 expression, it was undetermined whether this finding is true in lean and obese humans. Additionally, a more extensive evaluation of laminin-α chain expression had not been performed in mouse models of obesity. In this study, we confirm the correlation between obesity and increased LAMA4 in a mouse model and show that human sWAT displays a similar trend. Interestingly, we also note that weight loss does not appear to downregulate LAMA4 expression in humans or mice, at least in the short-term. *In vitro* findings demonstrated an increase in laminin-α chain expression in response to lipid loading and a potential association between a laminin-411 rich culture environment and lipid metabolism. Taken together, the weight loss and *in vitro* data suggest that the LAMA4-obesity connection is complex and is not modulated rapidly.

While the results of this study suggest that LAMA4 is significantly associated with obesity, we still do not fully understand the mechanisms underlying this observation. Prior publications have reported a role for LAMA4 in the inhibition of the thermogenic program in adipocytes, in regulating angiogenesis, and even in facilitating immune infiltration (23, 33, 34). There is a strong need to elucidate the role of LAMA4 in each of these processes in greater depth in human adipose, and to explore other pathways that may implicate LAMA4. In doing so, we hope to uncover how *LAMA4* may be targeted effectively in therapies for obesity.

## Data Availability Statement

The original contributions presented in the study are included in the article/**Supplementary Material**. Further inquiries can be directed to the corresponding author.

## Ethics Statement

The studies involving human participants were reviewed and approved by University of Chicago Institutional Review Board 09-337-B. The patients/participants provided their written informed consent to participate in this study. The animal study was reviewed and approved by University of Chicago Institutional Animal Care and Use Committee.

## Author Contributions

AG, AC, MB, and RC designed the experiments, which were performed by AG, AC, and LS. JW, MP, IC, and AL contributed to sample collection and preparation for all human samples. MG and EB provided expertise and consulted on experimental design and results. AG and AC performed all data analysis. AH and IC supported procedures related to adipose section staining and microscopy data analysis workflow. AG took lead in writing the manuscript with contributions from AC and LS. RC and MB supervised the project. All authors contributed to the article and approved the submitted version.

## Funding

This work was supported, in part, by the National Institutes of Health (Grant R01 DK 103014) and University of Chicago Diabetes Research and Training Center (National Institutes of Health Grant P30 DK020595). MG is supported by the National Institute of Diabetes and Digestive and Kidney Diseases of the National Institutes of Health, under Award Number F32-0DK122754.

## Conflict of Interest

The authors declare that the research was conducted in the absence of any commercial or financial relationships that could be construed as a potential conflict of interest.

## Publisher’s Note

All claims expressed in this article are solely those of the authors and do not necessarily represent those of their affiliated organizations, or those of the publisher, the editors and the reviewers. Any product that may be evaluated in this article, or claim that may be made by its manufacturer, is not guaranteed or endorsed by the publisher.
