# Supplementary material for: Laminin-α4 Is Upregulated in Both Human and Murine Models of Obesity
Source: Front Endocrinol (Lausanne). 2021 Jul 28;12:698621. doi: 10.3389/fendo.2021.698621 (PMC8355986; doi:10.3389/fendo.2021.698621)
Supplement: Supplementary file 2 [file DataSheet_2.docx]

Supplementary Material

# Supplementary Data

## ImageJ Macros utilized in immunofluorescence analysis and adipocyte size quantification

*Quantification of Extracellular LAMA4*

//pre-analysis//

Title=getTitle();

run("Set Measurements...", "area mean integrated limit display redirect=None decimal=0");

selectWindow(Title);

//split and rename channels//

run("Split Channels");

rename("DAPI");

selectWindow("C1-"+Title);

rename("Lama4");

selectWindow("Lama4");

run("Duplicate...", " ");

//Bright Spot Removal//

rename("BrightSpotsBinary");

setAutoThreshold("MaxEntropy dark");

run("Threshold...");

setOption("BlackBackground", false);

run("Convert to Mask");

selectWindow("Lama4");

run("Duplicate...", " ");

rename("Pre");

setOption("ScaleConversions", true);

run("8-bit");

imageCalculator("Subtract create", "Pre","BrightSpotsBinary");

rename("Pre-Background Removal");

//Background Removal//

run("Subtract Background...", "rolling=50 sliding");

rename(Title+"Lama4");

run("Duplicate...", " ");

//Threshold

setAutoThreshold("Triangle dark");

run("Threshold...");

run("Measure");

//cleanup//

run("Close All");

*Color Images Presentation of LAMA4*

//pre-analysis//

Title=getTitle();

selectWindow(Title);

//split and rename channels//

run("Split Channels");

rename("DAPI");

run("Subtract Background...", "rolling=30");

selectWindow("C1-"+Title);

rename("LAMA4");

run("Subtract Background...", "rolling=30");

run("Duplicate...", " ");

rename("LAMA4-Sub");

//Bright Spot Removal//

setAutoThreshold("Intermodes dark");

run("Threshold...");

setOption("BlackBackground", false);

run("Convert to Mask");

selectWindow("LAMA4");

setOption("ScaleConversions", true);

run("8-bit");

imageCalculator("Subtract create", "LAMA4","LAMA4-Sub");

//Brightness and Smoothing//

run("Brightness/Contrast...");

run("Enhance Contrast", "saturated=0.35");

run("Remove Outliers...", "radius=20 threshold=12 which=Bright");

run("Close");

rename("LAMA4-1");

//Color Channels//

selectWindow("DAPI");

setOption("ScaleConversions", true);

run("8-bit");

selectWindow("LAMA4-1");

run("Channels Tool...");

run("Green");

selectWindow("DAPI");

run("Channels Tool...");

run("Blue");

run("Merge Channels...", "c2=[LAMA4-1] c3=[DAPI] create keep");

run("Despeckle");

rename(Title+"Color Composite");

//Cleanup//

close("LAMA4")

close("DAPI")

close("LAMA4-Sub")

close("LAMA4-1")

*Measurement of Adipocyte Area*

Title= getTitle();

//enhance brightness//

run("Enhance Contrast", "saturated=0.35");

run("8-bit");

run("Invert");

run("Gaussian Blur...", "sigma=2");

run("Subtract Background...", "rolling=50");

setAutoThreshold("MinError dark");

run("Threshold...");

run("Convert to Mask");

run("Dilate");

run("Invert");

run("Shape Filter", "area=100-Infinity area_convex_hull=0-Infinity perimeter=0-Infinity perimeter_convex_hull=0-Infinity feret_diameter=0-Infinity min._feret_diameter=0-Infinity max._inscr._circle_diameter=0-Infinity area_eq._circle_diameter=0-Infinity long_side_min._bounding_rect.=0-Infinity short_side_min._bounding_rect.=0-Infinity aspect_ratio=1-Infinity area_to_perimeter_ratio=0-Infinity circularity=0-Infinity elongation=0-1 convexity=0-1 solidity=0-1 num._of_holes=0-Infinity thinnes_ratio=0-1 contour_temperatur=0-1 orientation=0-180 fractal_box_dimension=0-2 option->box-sizes=2,3,4,6,8,12,16,32,64 draw_holes");

run("Invert");

run("Analyze Particles...", "size=500-Infinity show=Outlines display exclude");

# Supplementary Figures and Tables

## Supplementary Tables

| **Species** | **Forward** | **Reverse** |
| --- | --- | --- |
| **Human** |  |  |
| LAMA2 | Qiagen - PPH21264E-200 |  |
| LAMA4 | GACCCTGAGGACACAGTGTTTT | AGGCAGGTTTAAGCTGGTAGG |
| LAMA5 | Qiagen - PPH07801A-200 |  |
| COL6A3 | Qiagen - PPH00843B-200 |  |
| COL4A1 | Qiagen - PPH20687A-200 |  |
| COL3A1 | CTTCTCTCCAGCCGAGCTTC | GACCCCATCAGCTTCAGG |
| COL1A1 | AGCGTGGCCTACATGGAC | CGACAGTGACGCTGTAGGTG |
| GAPDH | TGC ACC ACC AAC TGC TTA GC | GGC ATG GAC TGT GGT CAT GAG |
| RPL13α | AAG GTC GTG CGT CTG AAG | GAG TCC GTG GGT CTT GAG |
| YWHAZ | TGC TTG CATCCC ACA GAC TA | AGGCAGACAATGACAGACCA |
|  |  |  |
| **Mouse** |  |  |
| GAPDH | CAATGTGTCCGTCGTGGATCTGA | GAGTTGCTGTTGAAGTCGCAGGA |
| LAMA1 | CAGCGCCAATGCTACCTGT | GGATTCGTACTGTTACCGTCACA |
| LAMA2 | TCCCAAGCGCATCAACAGAG | CAGTACATCTCGGGTCCTTTTTC |
| LAMA3 | CTGTGACTACTGCAATTCTGAGG | CAAGGTGAGGTTGACTTGATTGT |
| LAMA4 | GGAATACCTGAACGTGCACATGAGA | GTGCCATCTGCCATCACAGAGATTCT |
| COL1A1 | TCCGGCTCCTGCTCCTCTTA | GTATGCAGCTGACTTCAGGGATGT |
| COL3A1 | GCCCACAGCCTTCTACAC | CCAGGGTCACCATTTCTC |
| COL4A1 | AGGATGCAACGGTACAAAGGGAGA | TGGCCGAGAATTTCACCAGGATCT |
| COL6A3 | GAACCACGGAAGAGAGCAAG | CGGCTTCACATCAAGTTCCT |
| ADIPOQ | TGTTCCTCTTAATCCTGCCCA | CCAACCTGCACAAGTTCCCTT |
| PPARg | TGCAGGTTCTACTTTGATCGC | CTGCTCCACACTATGAAGACAT |
| FABP4 | ACACCGAGATTTCCTTCAAACTG | CCATCTAGGGTTATGATGCTCTTCA |
| PLIN1 | Qiagen - PPM35320A-200 |  |
| ACOX-1 | TCGAAGCCAGCGTTACGAG | ATCTCCGTCTGGGCGTAGG |
| CPT1a | CTATGCGCTACTCGCTGAAGG | GGCTTTCGACCCGAGAAGA |
| CPT1b | TGGGACTGGTCGATTGCATC | TCAGGGTTTGTCGGAAGAAGAA |
| ATGL | CCAACGCCACTCACATCTAC | GCCTCCTTGGACACCTCAAT |
| PPARα | TCGGCGAACTATTCGGCTG | GCACTTGTGAAAACGGCAGT |

**Supplementary Table 1.** Primers used in Quantitative Real-Time PCR.

|  | **Control** | **Obese Pre-Surgery** | **Obese Post-Surgery** |
| --- | --- | --- | --- |
| **Subjects** | 3 | 9 | 6 |
| **Weight (kg)** | 64.13 (+ 8.12) | 123.31 (+ 10.89) | 102.96 (+ 16.70) |
| **Height (cm)** | 161.76 (+ 7.35) | 166.89 (+ 6.26) | 165.4 (+ 6.64) |
| **BMI (kg/m^2)** | 24.53 (+ 2.59) | 44.26 (+ 3.05) | 37.73 (+ 6.72) |
| **Age** | 39 (+ 16.52) | 33 (+ 7.62) | 39 (+ 4.94) |

**Supplementary Table 2.** Human Study Subject Characteristics (Immunofluorescence Experiments).


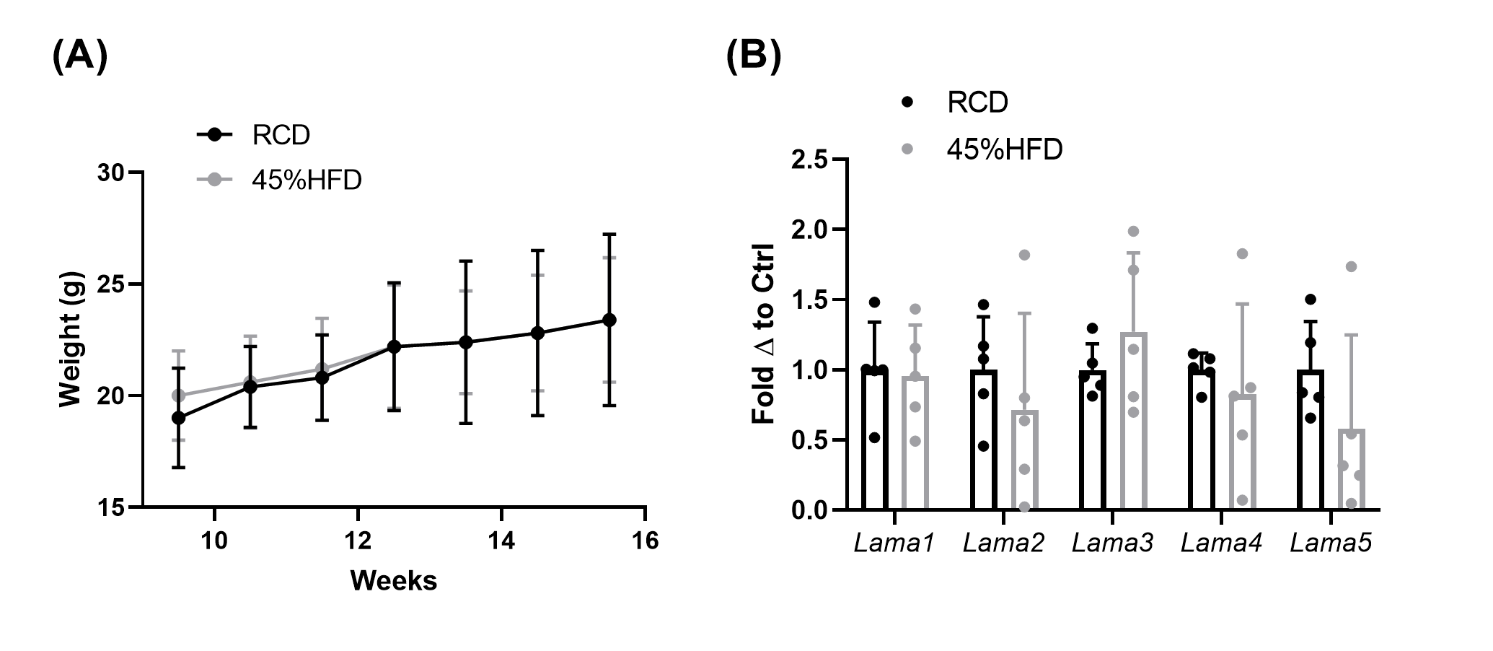
**Supplementary Figure 1.** Female mice dietary study and laminin expression

1. Weights of female mice placed on 45% HFD or chow (RCD) diet for 8 weeks. HFD (n=5), RCD (n=5).
2. Laminin alpha chain mRNA expression in sWAT from female mice on 45% HFD or RCD for 8 weeks. HFD (n=5), RCD (n=5).

**
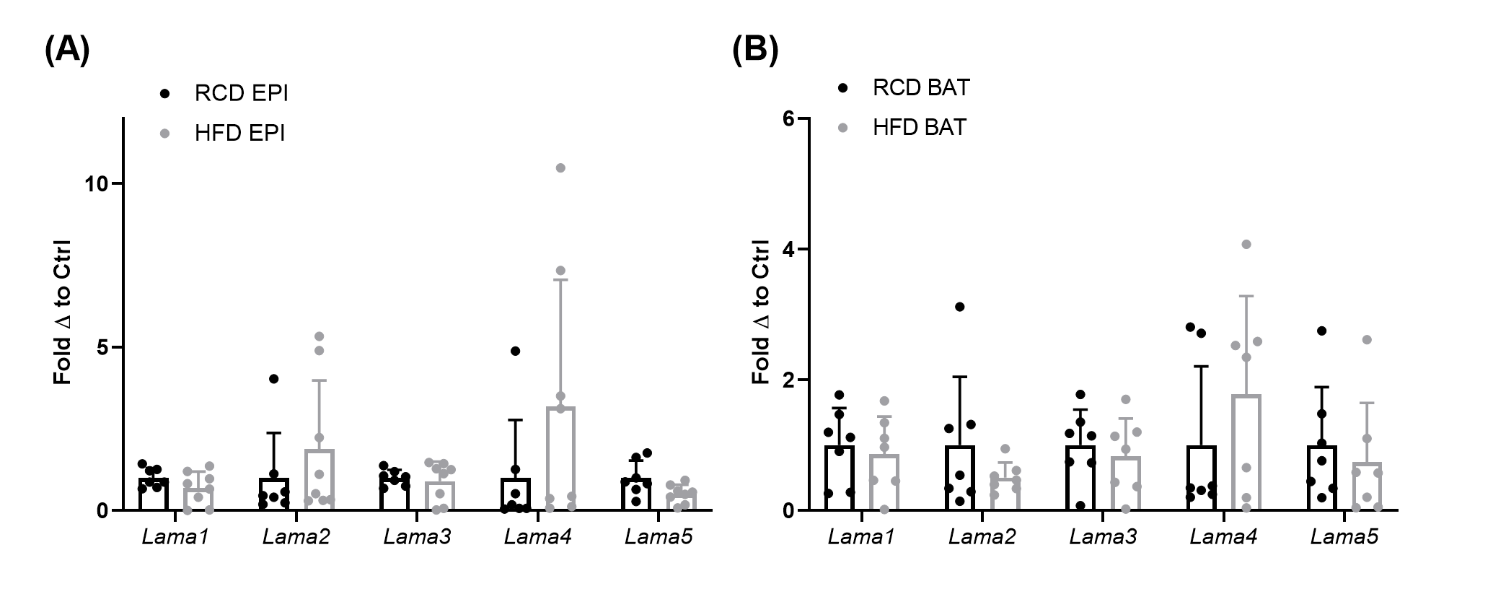
**

**Supplementary Figure 2.** Laminin expression in other adipose depots from male mouse dietary studies

**(A)** Laminin alpha chain mRNA expression in Epididymal WAT (EPI) from male mice on 45% HFD or RCD for 8 weeks. HFD (n=8), RCD (n=7).

**(B)** Laminin alpha chain mRNA expression in Brown Adipose Tissue (BAT) from male mice on 45% HFD or RCD for 8 weeks. HFD (n=7), RCD (n=7).

**
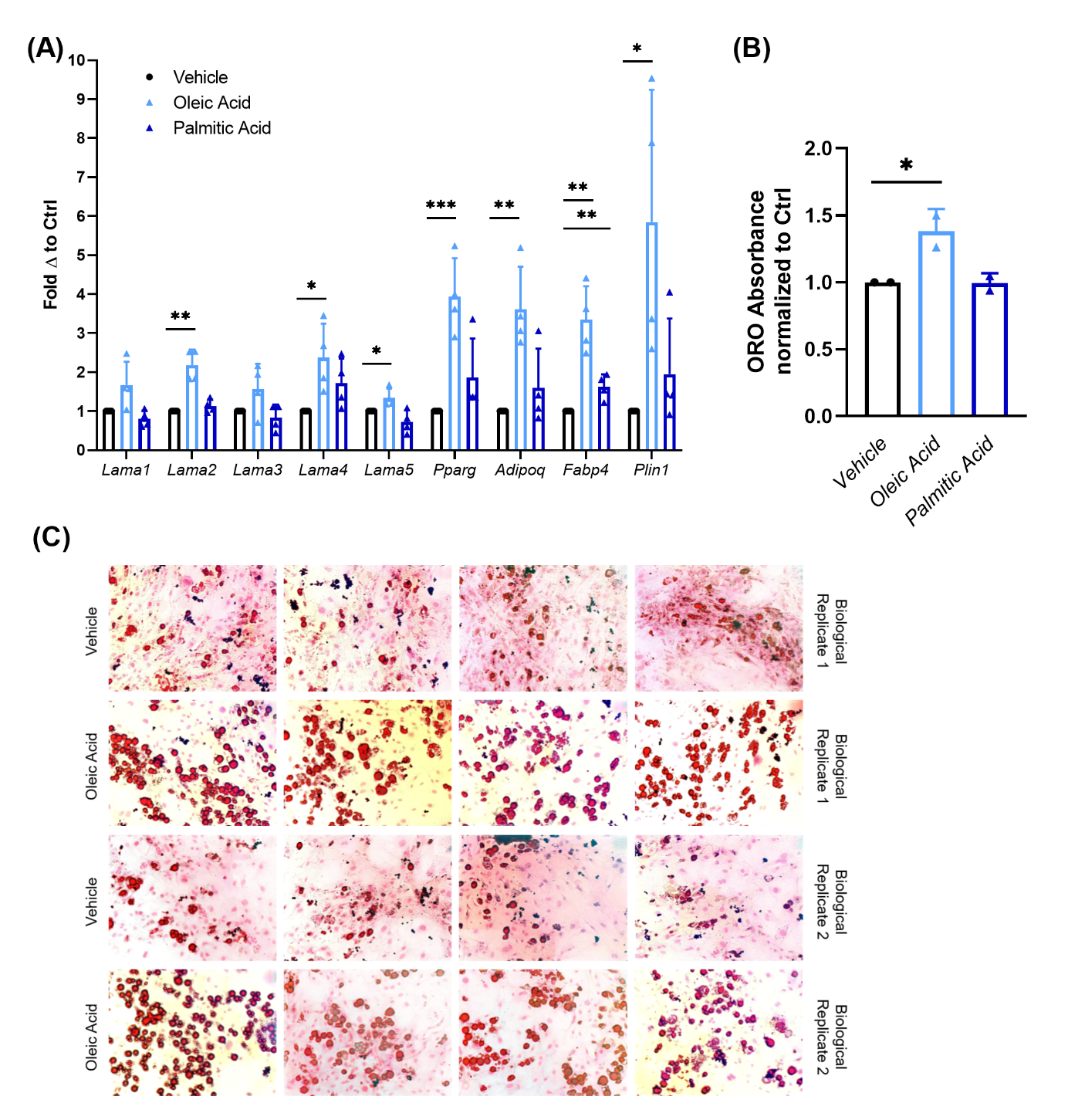
**

**Supplementary Figure 3.** Effect of different fatty acid treatments during differentiation of adipocytes

**(A)** Laminin alpha chain mRNA expression and adipogenic gene expression in differentiated murine primary adipocytes treated with Vehicle, 0.25 mM oleic acid, or 0.25 mM palmitic acid for four days. Veh (n=4), Oleic Acid (n=4), Palmitic Acid (n=4).

**(B)** Semi-quantitative measurement of lipid content by ORO absorbance at 492 nm, normalized to Vehicle. Veh (n=2), Oleic Acid (n=2), Palmitic Acid (n=2).

**(C)** Images of ORO stained adipocytes, 40X. Veh (n=2), Oleic Acid (n=2).

**
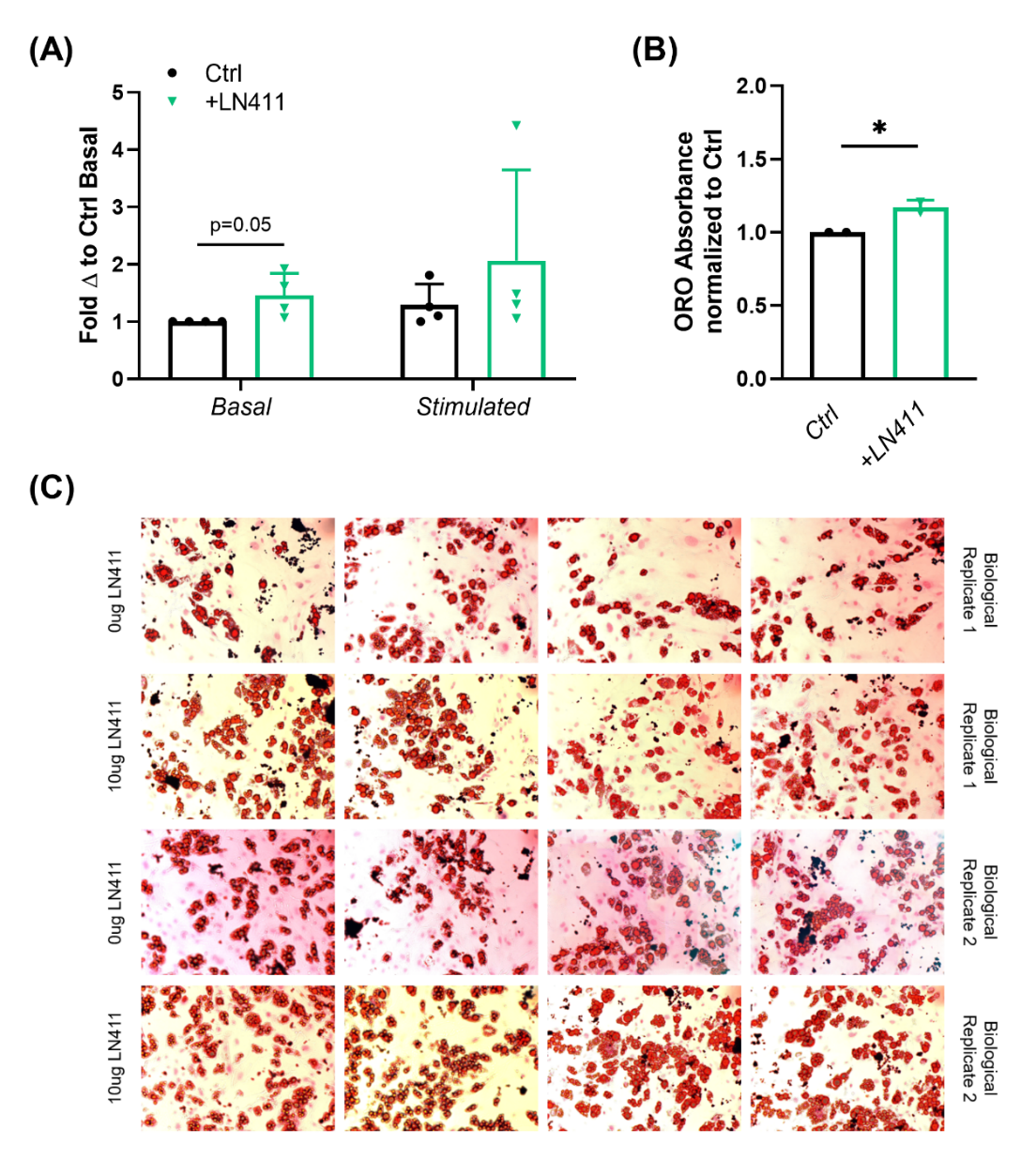
Supplementary Figure 4.** Lipolysis and lipid accumulation in response to LN411 treatment

**(A)** Relative lipolysis activity, as measured by glycerol content, in differentiated murine primary adipocytes grown on culture plate coated with or without LN411. Basal lipolysis indicates wells where no isoproterenol was added, while stimulated lipolysis indicates wells where isoproterenol was added. Basal (n=4), Stimulated (n=4).

**(B)** Semi-quantitative measurement of lipid content by ORO absorbance at 492 nm, normalized to ctrl. Ctrl (n=2), +LN411 (n=2).

**(C)** Images of ORO stained adipocytes, 40X. Ctrl (n=2), 10ug LN411 (n=2).

# Original Blot Images


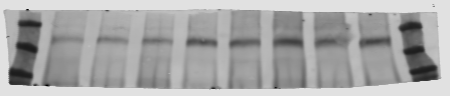

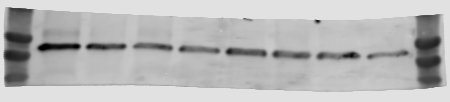


HFD

RCD

250

150

50

37

Original blot image for Figure 1 showing LAMA4 expression (202kDa - Invitrogen MA5-24651) and Beta-Actin expression (45kDa – CST 4970) from SQ protein lysates of male mice.
